# RadCoT: a Radiological Chain-of-Thought framework for enhanced error detection in radiology reports

**DOI:** 10.1186/s41747-026-00804-0

**Published:** 2026-09-22

**Authors:** Jia Li, Zichun Zhou, Yantao Niu, Pengfei Zhao, Yan Xu, Xinghao Wang, Lihua Wang, Lining Dong, Wei Wei, Xuan Wei, Zhenchang Wang, Han Lv

**Affiliations:** 1 Department of Radiology, Beijing Friendship Hospital, Capital Medical University, 95 Yong’an Road, Xicheng District, Beijing, 100050 China; 2 Department of Radiology, Shengjing Hospital of China Medical University, Beijing, People’s Republic of China; 3 Institute for Medical Informatics, University of Luebeck, Luebeck, Germany; 4 Medical Digital Intelligence Innovation Center, Beijing Friendship Hospital, Capital Medical University, 95 Yong’an Road, Xicheng District, Beijing, 100050 China; 5 Precision and Intelligence Medical Imaging Lab, Beijing Friendship Hospital, Capital Medical University, Beijing, China; 6 Department of Health Data Science, School of Medical Technology, Capital Medical University, Beijing, China

**Keywords:** Artificial intelligence, Diagnostic errors, Natural language processing, Radiology

## Abstract

**Background:**

Errors in radiology reports are a major patient-safety concern and are difficult to detect with manual quality assurance (QA). Large language models (LLMs) can assist, but generic prompting does not reflect radiologists’ structured, section-based workflows.

**Objective:**

To develop and evaluate RadCoT (Radiological Chain-of-Thought), a domain-specific prompting framework aligning LLM reasoning with radiological review workflows, and to assess whether it enables open-source models to approach commercial benchmarks for error detection.

**Materials and methods:**

In this retrospective study, 1,170 clinician-validated errors were extracted from a departmental QA repository (January 2021–December 2024), corresponding to 900 error-containing reports. An additional 300 error-free reports served as controls, yielding 1,200 reports balanced across radiography, ultrasound, CT, and MRI. Errors were categorized into five types by experienced radiologists. Seven LLMs were evaluated using standard prompting and the six-step RadCoT framework. Micro-averaged precision, recall, and F1 were computed at the error-instance level. Error type, modality-specific performance, and inference time were analyzed.

**Results:**

RadCoT significantly improved the mean micro-averaged F1 across all models from 0.77 ± 0.06 (standard) to 0.85 ± 0.06 (RadCoT; *p* = 0.003). GPT-4o with RadCoT achieved the highest F1 (0.93). Llama-3.3-70B with RadCoT (F1 = 0.89) narrowed the gap with GPT-4o under standard prompting (F1 = 0.88; paired *t*-test, Holm-adjusted *p* = 0.28). Interpretation errors showed the largest gain, with F1 improving from 0.57 to 0.75.

**Conclusion:**

RadCoT consistently enhances LLM-based error detection, particularly for complex logic and consistency errors, closing the gap between open-source and commercial models and offering a pathway for privacy-preserving, on-premises QA.

**Key Points:**

***Question*** Manual review misses radiology report errors and remains difficult to scale.

***Findings*** Structured prompting improves detection of interpretation and section-consistency errors across seven models.

***Relevance statement*** Open-source models approach commercial performance for privacy-preserving on-premises radiology quality assurance.

**Graphical Abstract:**

**Figure:**
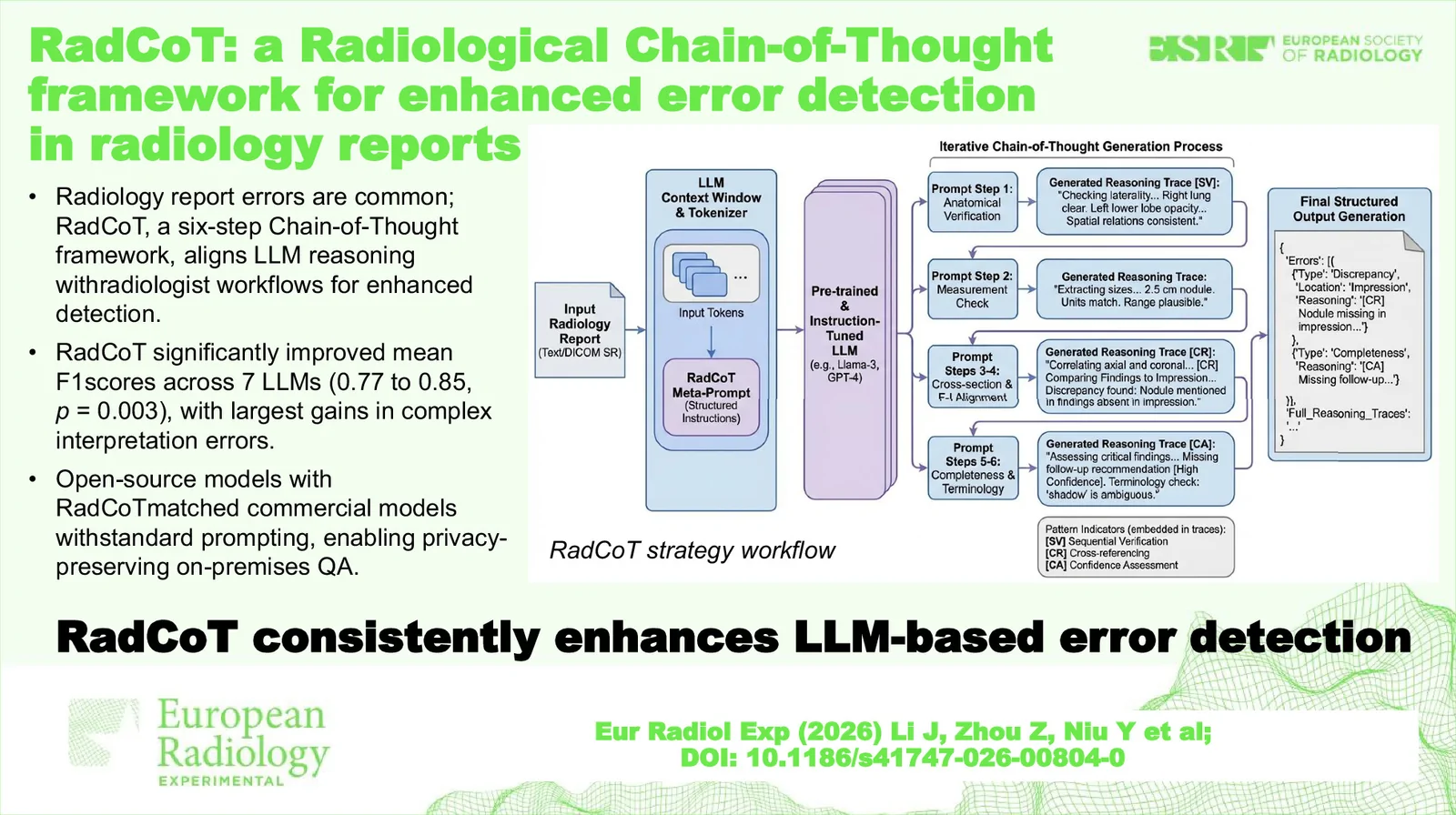

## Introduction

Radiology reports are the primary communication channel between radiologists and referring clinicians, yet clinically important errors remain common. Prior work has reported overall error and discrepancy rates between 2% and 20%, these errors span a broad spectrum—from formal reporting defects (*e.g.*, typographical or unit inconsistencies) that carry little direct clinical consequence, to interpretive discrepancies (*e.g.*, missed critical findings, incorrect diagnostic conclusions) that may directly affect patient management. [1–4]. Many of these errors are interpretive rather than perceptual, arising from cognitive biases, fatigue, and communication lapses [1, 2]. Traditional QA programs rely on selective peer review, discrepancy conferences, and audit meetings, which are resource-intensive and increasingly strained by rising imaging volumes and workforce shortages [5–7].

Radiology reports differ from general clinical text through their standardized structure, modality-specific terminology, and emphasis on internal consistency. Best-practice guidelines emphasize clear organization into clinical information, technique, findings, and impression, as well as unambiguous anatomy, laterality, and measurements [8, 9]. Surveys of radiologists and referring clinicians highlight persistent dissatisfaction with the clarity, completeness, and consistency of reports despite the widespread use of templates and structured reporting tools [10, 11]. Pre-populated templates can reduce turnaround time but may introduce systematic copy-forward errors and overlooked template elements if not carefully reviewed [11].

Recent advances in LLMs have created new opportunities for scalable QA in radiology. GPT-4 and related models have shown strong performance in transforming free-text reports into structured templates, with near-perfect fidelity in some settings [12]. LLMs can match radiologists in detecting deliberately inserted errors in neuroradiology and mixed-modality reports, while operating at a fraction of the time and cost [13, 14]. Parallel work has yielded expert-annotated datasets, such as ReXVal, that capture clinically significant and insignificant errors in generated reports, enabling benchmarking of medical text generation and evaluation [15]. Structured reporting templates, checklist-driven QA approaches, and contextual reporting frameworks already impose a form of sequential, section-based cognitive review during report creation and have been shown to reduce omissions and improve guideline adherence [16–18]. Nevertheless, few automated methods have extended this section-aware reasoning to retrospective QA of already finalized free-text reports.

At the same time, radiology-focused LLM studies have begun to explore task-specific prompting and CoT strategies. Park et al evaluated context-aware prompting for classifying tumor response categories from spine MRI reports, showing that in-context learning combined with CoT improves AUROC and calibration for tumor response classification [19]. Takita et al compared Claude, GPT, and Gemini in structuring head CT reports for intracranial hemorrhage and skull fractures, demonstrating that prompting strategy modulates performance but that all combinations achieve high accuracy [20]. Prucker et al used locally deployed Llama models with CoT prompting to generate structured lymphoma progression reports from cross-sectional imaging in a privacy-preserving setting, achieving high accuracy for data extraction but lower accuracy for derived staging and treatment response [21]. Beyond radiology, Mahmoudi et al reported near-perfect performance of open-source LLMs for automated echocardiography report interpretation using instruction-tuned prompts with and without CoT [22], and Kim et al fine-tuned a generative LLM with CoT to infer pathologic TN classification and rationales from lung cancer pathology reports [23]. Xiong et al further extended prompt engineering to multimodal GPT-4o for panoramic dental radiographs, integrating CoT and multimodal in-context learning in a meta-prompt to improve error localization and reasoning [24].

CoT prompting, which encourages models to generate intermediate reasoning steps, has emerged as a powerful strategy for complex reasoning tasks [25]. Domain-adapted CoT approaches have been explored in medical coding, diagnostic reasoning, and clinical information extraction, where they can enhance both performance and interpretability and can partially compensate for a smaller model size or limited training data [26–29]. At the same time, foundation models raise concerns about hallucinations, bias, and alignment with domain-specific safety norms, underscoring the need for task-tailored guardrails and workflows [30]. Although some radiology studies already exploit CoT for classification or staging [19, 21, 22], there has been little systematic work on tailoring CoT frameworks specifically to radiology report QA or on analyzing how emergent reasoning patterns relate to error-detection performance.

In this study, we introduce RadCoT (Radiological Chain-of-Thought), a six-step prompting framework designed to mirror expert radiologist review of structured reports. RadCoT guides LLMs through sequential checks of anatomy, measurements, intersection consistency, findings-impression alignment, clinical completeness, and terminology. Using a large library of real-world radiology report errors extracted from a departmental QA repository, we compare RadCoT with generic COT and standard prompting across seven contemporary LLMs, including both commercial and open-source models.

## Materials and methods

### Study design and dataset construction

The institutional review board of Beijing Friendship Hospital, Capital Medical University, waived written informed consent (approval/waiver ID: 2021-P2-142-01) because all reports were de-identified and the study posed minimal risk.

We leveraged an existing departmental QA repository that prospectively captures errors identified during routine clinical practice and peer review, in line with contemporary QA frameworks. From this repository, we extracted 1,170 clinician-validated errors recorded between January 2021 and December 2024. Each QA entry was linked to the corresponding finalized radiology report. As a result, the 1,170 errors were mapped to 900 unique reports containing errors. For negative controls, we selected an additional 75 error-free reports per modality from the same time period. Each candidate's control was independently reviewed and cross-validated by two senior radiologists with 10 years’ experience to confirm the absence of textual errors.

Most error-containing reports had a single annotated error; the distributions across modalities differed significantly, and CT/MRI errors were more common. The overall median was 1 (IQR 1–2). CT and MRI reports exhibited higher mean errors per report than radiography and ultrasound reports, consistent with their greater complexity and prior observations in cross-sectional imaging [2, 20, 23]. The QA database interface is shown in Supplementary Material Fig. SP1. This study was reported in accordance with the REFINE international consensus guideline [31] for reporting on the use of foundation models in healthcare; the completed REFINE checklist is provided as Supplementary Table S2.

### Error definition and annotation

For this study, an additional independent re-review was performed: three board-certified radiologists (10, 15, and 18 years of experience) independently re-reviewed each report-error pair. Each error was assigned to one of five mutually exclusive categories, adapted from prior literature on diagnostic and reporting errors. Numerical errors involve incorrect measurements, units, or internally inconsistent quantitative values; for instance, erroneously recording a dimension in meters rather than millimeters or decimeters. Omission/insertion errors reflect the mention of structures or findings that are not actually present or the failure to mention present ones. A striking example includes describing the appearance of a uterus in an abdominal CT report for a male patient. Interpretation errors result in incorrect diagnostic conclusions or misjudgments about severity or clinical importance. For example, a chest CT description notes a large pulmonary mass, yet the impression erroneously suggests “observation” rather than immediate intervention. Findings-impression discrepancies capture inconsistencies between the detailed findings and the final conclusion, including omitted key findings or contradictory statements. Common instances include a hepatic cyst described in the findings but absent from the impression, or an impression suggesting “possible ischemic changes” when the findings section explicitly describes the brain parenchyma as normal. Data example is shown in Table SP2.

After independent annotation, disagreements were resolved by consensus. Residual disagreements (4.5% of instances) were adjudicated by a senior radiologist with 20 years of experience. Inter-rater agreement before consensus was substantial, with a Cohen’s *κ* of 0.78. The final error-type distribution is shown in Table 1, with complex interpretive categories (interpretation, omission/insertion, and findings–impression discrepancies) comprising 68.4% of all errors.

**Table 1:** Distribution of error types in the reference library

Error type	Definition (summary)	Errors	Percentage of all errors (%)
		(*n*)	
Typographical	Spelling, punctuation, or minor grammar issues without a change in clinical meaning	180	15.4
Numerical	Incorrect measurements, units, or inconsistent quantitative values	190	16.2
Omission/insertion	Missing critical findings or mention of unsupported findings	260	22.2
Interpretation	Incorrect diagnostic conclusions or misjudged severity/significance	310	26.5
Findings–impression discrepancy	Inconsistencies between the findings section and the impression	230	19.7
Total		1,170	100

To contextualize likely deployment performance beyond the error-enriched evaluation set, we conducted a simple prevalence simulation assuming clinical error rates of 2%, 5%, 10%, and 20%. Using an illustrative deployment-oriented operating point derived from the observed miss rate and false-positive frequency in the present study (sensitivity = 0.75; specificity = 0.91), prevalence-specific positive predictive value (PPV) and negative predictive value (NPV) were estimated using Bayes’ theorem: PPV = Se × Prev/[Se × Prev + (1 − Sp) × (1 − Prev)] and NPV = Sp × (1 − Prev)/[(1 − Se) ×  Prev + Sp × (1 − Prev)]. The expected numbers of false positives, false negatives, and flagged reports per 1,000 cases were then calculated as (1 −  Sp) × (1 − Prev) × 1,000, (1 − Se) ×  Prev  × 1,000, and [Se × Prev  + (1 − Sp) × (1 − Prev)] × 1,000, respectively.

### Large language models and implementation

We evaluated seven LLMs representing both commercial and open-source families. Commercial models included GPT-4o (OpenAI) and Claude-3.5 Sonnet (Anthropic), both accessed via HTTPS APIs with institutionally approved data-handling agreements. Open-source models included Llama-3-70B and Llama-3.3-70B (Meta), Mixtral-8×22B (Mistral AI), Gemma-2-27B (Google), and Qwen-2.5-72B (Alibaba). Model sizes ranged from 27 to approximately 200 billion parameters, with context windows between 8k and 200k tokens, in line with recent foundation-model deployments [10, 24]. Open-source models were hosted on an on-premises GPU server equipped with NVIDIA V100-class hardware. All model execution occurred within the institutional network boundary to maintain data privacy and comply with local data-governance policies. To ensure reproducibility and fair comparison, we applied uniform decoding settings across all models: temperature = 0 (greedy decoding) and maximum output length = 4,096 tokens(no outputs reached this limit in our experiments). For open-source models, the repetition penalty was set to 1.0 (no penalty). The GPT-4o API does not expose a unified repetition penalty but offers frequency_penalty and presence_penalty, both of which were set to 0 (default); the Claude-3.5 Sonnet API does not expose equivalent penalty parameters. Each report was processed once under identical settings for both prompting strategies [13, 14].

### Prompting strategies

Each report was processed independently under two prompting strategies applied to every model. Prompts were phrased in English and included explicit instructions to avoid adding or removing clinical content beyond error identification. The RadCoT reasoning steps were specified a priori based on the established radiological diagnostic workflow and were frozen prior to evaluation; no prompt modifications were made in response to performance on the evaluation corpus.

#### **Standard prompting**

The model received a single direct instruction to read the report and list any errors with brief explanations, without explicit guidance on intermediate reasoning. This setting approximates how LLMs are often used informally by clinicians today [32]. The complete verbatim text of both prompts—including the system role, step-level instructions for all six RadCoT stages, and the required structured output schema—is provided in Supplementary Document S1 and mirrored in the accompanying GitHub repository.

#### **RadCoT prompting**

**Fig. 1:**
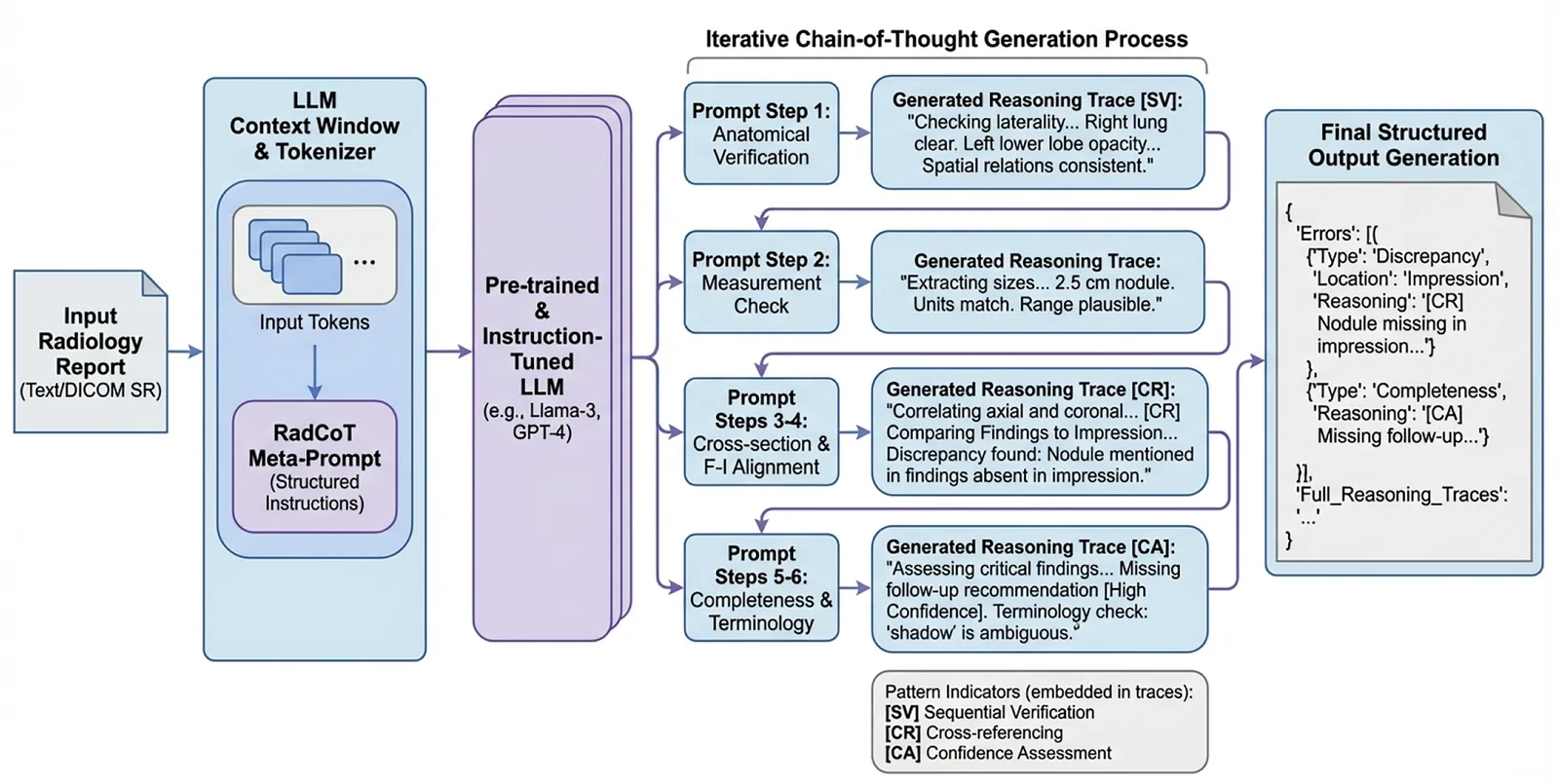
RadCoT prompt strategy workflow

**Fig. 2:**
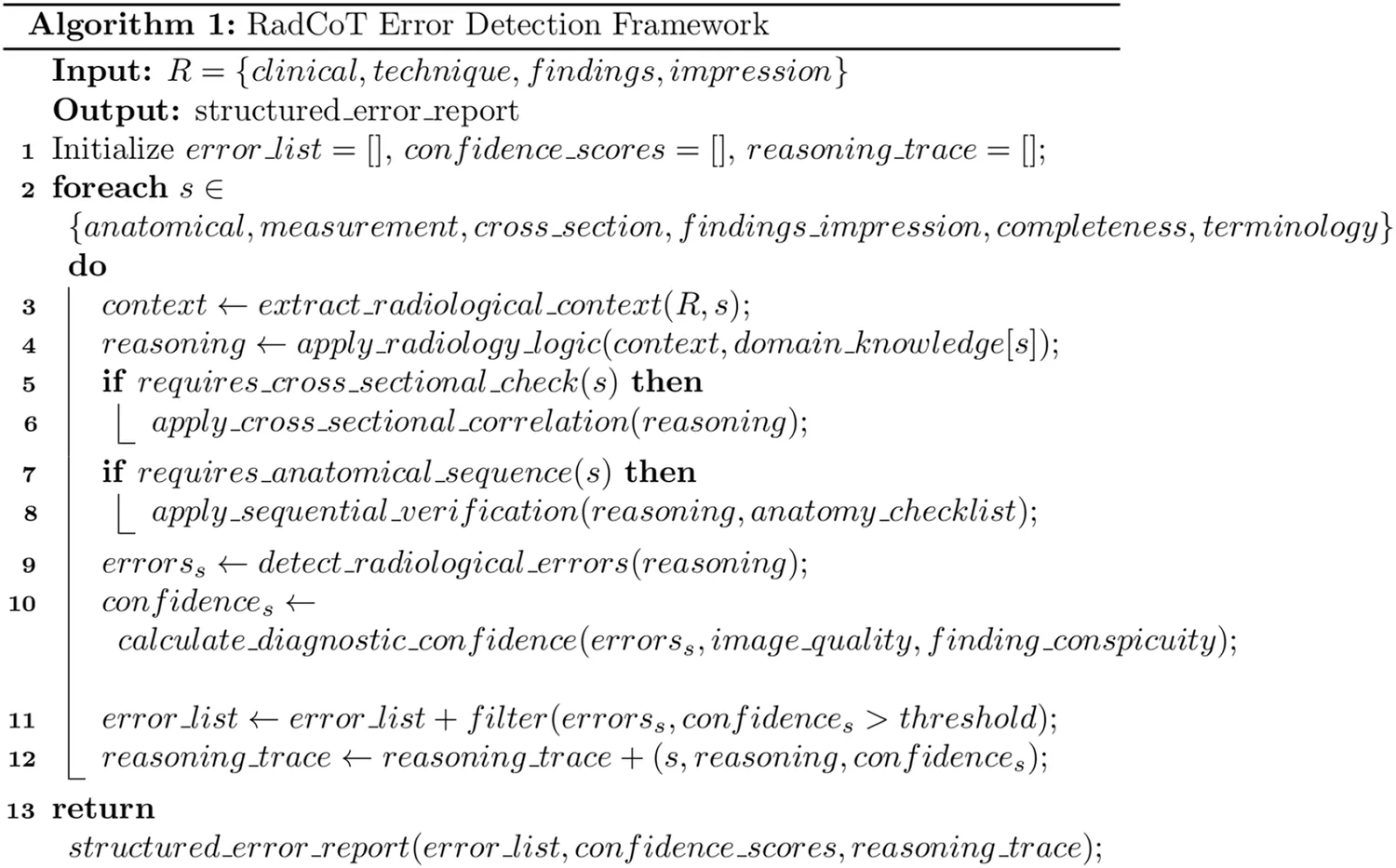
Algorithm 1. Pseudocode of RadCOT

The model was guided through a six‑step, radiology‑specific reasoning framework. The prompt outlined an ordered review of anatomical correctness, measurement consistency, intersection correlation, findings–impression alignment, clinical completeness, and terminology accuracy. The final instruction asked the model to output a structured list of errors along with the reasoning steps that led to each item. The RadCoT prompt strategy is shown in Fig. 1. The pseudocode used in RadCoT is depicted in Fig. 2: **Algorithm 1**.

In addition to the original standard-prompting and RadCoT experiments, we conducted an analysis comparing a simple domain-neutral generic chain-of-thought (CoT) prompt (Supplementary Document S6) with RadCoT in GPT-4o and Llama-3.3-70B. The generic CoT condition instructed the model to reason step by step before listing report errors, without the radiology-specific section-level scaffold used in RadCoT.

### RadCoT framework

RadCoT is designed to approximate expert radiologist review workflows described in the quality-assurance literature [8]. In the first step, the model verifies anatomical terms, laterality, and spatial relationships to identify mismatched organs, wrong-side references, or impossible configurations. The second step focuses on measurements, ensuring internal consistency of lesion sizes, units, and multiplicity across sections. The third step explicitly prompts the model to cross-reference information across report sections, imaging planes, and sequences where applicable, to identify internal inconsistencies.

In the fourth step, the model aligns detailed findings with the impression, checking that key lesions, measurements, and qualifiers (for example, “possible”, “suspicious for”, “no definite”) are correctly summarized. The fifth step assesses clinical completeness by determining whether any critical structures or expected follow-up recommendations are missing, based on the described clinical scenario and the modality-specific checklists. In the final step, the model verifies that radiological terminology follows standardized usage (for example, RadLex-like descriptors) and avoids ambiguous or obsolete expressions.

### Outcome definition and statistical analysis

#### Reference standard

For each report, the reference standard was the set of clinician-validated error instances extracted from the QA repository and re-adjudicated by board-certified radiologists, with each instance assigned an error category and anchored to the corresponding sentence-level text span. Model outputs were normalized into discrete candidate error items (error type +  supporting snippet/section). Each candidate was matched to the reference set using a pre-specified rule combining sentence-level localization (same or immediately adjacent sentence) and semantic equivalence, the latter assessed via a locally trained sentence-matching model previously developed and validated by our group for radiology text [33]. All automated matches were independently verified by two board-certified radiologists blinded to model identity, with disagreements adjudicated by a senior radiologist.

A prediction was counted as a true positive (TP) if it (i) localized the error within the same sentence or an immediately adjacent sentence and (ii) assigned the correct error category. Unmatched predictions were counted as false positives (FP), and reference errors without a matched prediction were counted as false negatives (FN).

#### Primary metrics

Performance was evaluated at the error-instance level using micro-averaging across the full corpus. Precision (*P*), recall (*R*), and F1 were computed as:

$${{{\rm{P}}}}=\frac{{{{\rm{TP}}}}}{{{{\rm{TP}}}}+{{{\rm{FP}}}}}{{{\rm{R}}}}=\frac{{{{\rm{TP}}}}}{{{{\rm{TP}}}}+{{{\rm{FN}}}}}$$

$${{{\rm{F}}}}1=\frac{2{{{\rm{PR}}}}}{{{{\rm{P}}}}+{{{\rm{R}}}}}=\frac{2{{{\rm{TP}}}}}{2{{{\rm{TP}}}}+{{{\rm{FP}}}}+{{{\rm{FN}}}}}.$$

We additionally reported category-specific and modality-specific F1 scores by pooling error instances within each subgroup and applying micro-averaging within that subgroup. To reflect real-world constraints, open-source models were executed in a shared GPU environment with clinical-priority scheduling, which can increase latency relative to dedicated benchmarking; however, both prompting strategies were evaluated under identical execution conditions. All analyses were performed in Python (SciPy/scikit-learn); a corrected two-sided *p* < 0.05 was considered statistically significant. Multiple comparisons across the pre-specified family of *m* = 20 pairwise model × prompting-strategy tests per metric were controlled using the Holm–Bonferroni procedure (family-wise *α* = 0.05); all reported *p*-values are Holm-adjusted.

## Results

### Dataset characteristics

The 1,200-report corpus included 300 reports each for radiography, ultrasound, CT, and MRI. Among the 900 error-containing reports, radiography and ultrasound reports tended to be shorter and less complex than CT and MRI reports, with median lengths of 210 and 260 words *versus* 340 and 380 words, respectively. Overall dataset statistics are shown in Table 2.

**Table 2:** Characteristics of the evaluation dataset

Modality	Total reports	Error-containing reports	Control reports	Total errors	Median errors per error-containing report (IQR)^a^
	(*n*)	(*n*)	(*n*)	(*n*)	
Radiography	300	225	75	240	1 (1–1)
Ultrasound	300	225	75	255	1 (1–2)
CT	300	225	75	330	1 (1–2)
MRI	300	225	75	345	1 (1–3)
All	1,200	900	300	1170	1 (1–2)

^a^ Median errors were computed among error-containing reports only

Of the 1,170 errors, 180 (15.4%) were typographical, 190 (16.2%) numerical, 260 (22.2%) omission/insertion, 310 (26.5%) interpretation, and 230 (19.7%) findings–impression discrepancies (Table 1). Thus, nearly seven in ten errors involved higher-level interpretive or cross-sectional reasoning. Interpretation errors were particularly frequent in CT and MRI, whereas typographical and numerical errors were more common in radiography and ultrasound reports, consistent with prior observations that complex cross-sectional imaging generates more interpretive challenges. The distribution of errors per report was strongly skewed toward single-error cases, with 75.7% of error-containing reports containing exactly one error and only 4.6% containing three or more (Supplementary Table S4).

### Overall model performance

**Fig. 3:**
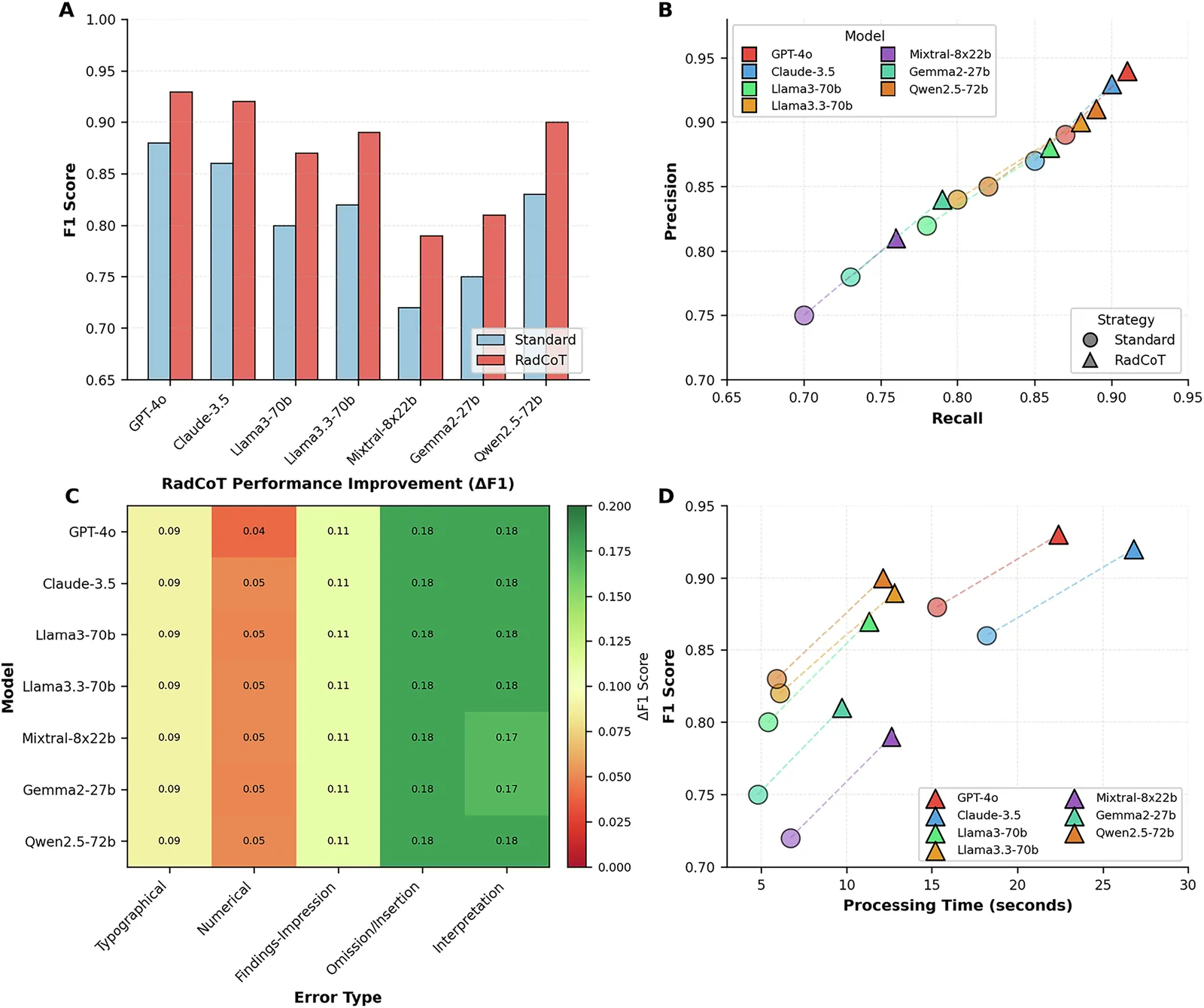
Overall model performance and computational trade‑offs for standard prompting *versus* RadCoT. **A** Micro‑averaged F1 by model, standard prompting *versus* RadCoT. **B** Precision–recall shift with RadCoT across models. **C** Heatmap of F1 gain (ΔF1) with RadCoT by error type and model. **D** F1 *versus* per‑report inference time (accuracy–efficiency)

Figure 3 summarizes detection performance and efficiency across models under standard prompting *versus* RadCoT. Averaged over all seven models, RadCoT increased micro-averaged F1 from 0.77 ± 0.06 to 0.85 ± 0.06 (mean difference 0.08; 95% CI, 0.06–0.10; *p* = 0.003), indicating a consistent benefit across model families.

Among commercial systems, GPT-4o achieved the highest F1 with RadCoT (0.93, 95% CI 0.88–0.94), improving from 0.88 under standard prompting, and Claude-3.5 Sonnet improved from 0.86 to 0.92 (*p* = 0.009). Open-source models showed comparable gains with RadCoT, reaching F1 scores of 0.90 (Qwen-2.5-72B), 0.89 (Llama-3.3-70B), 0.87 (Llama-3-70B), 0.86 (Mixtral-8×22B), and 0.82 (Gemma-2-27B) (Fig. 3A). Llama-3.3-70B + RadCoT (0.89) approached GPT-4o under standard prompting (0.88), although with no statistically significant difference on this dataset (paired *t*-test, Holm-adjusted *p* = 0.28).

RadCoT improved the precision–recall balance across all models (Fig. 3B), shifting points toward the upper-right: mean precision increased from 0.80 to 0.88 and mean recall from 0.75 to 0.83, consistent with simultaneous reductions in false positives and false negatives. These gains came with a modest runtime cost (Fig. 3D): RadCoT increased per-report inference time by 68.2%, largely due to longer reasoning traces. For GPT-4o, inference time rose from 15.3 ± 2.3 s to 22.4 ± 3.4 s, while open-source models required 9.7–12.8 s/report with RadCoT. Even so, processing remained well below typical manual review times (3–8 min/report) and was stable across modalities. supporting use in retrospective QA and pre-sign-off review workflows where per-report latency on the order of seconds is acceptable. In resource-constrained settings, Gemma-2-27B + RadCoT offered a favorable balance, achieving F1 = 0.82 in < 10 s/report.

**Fig. 4:**
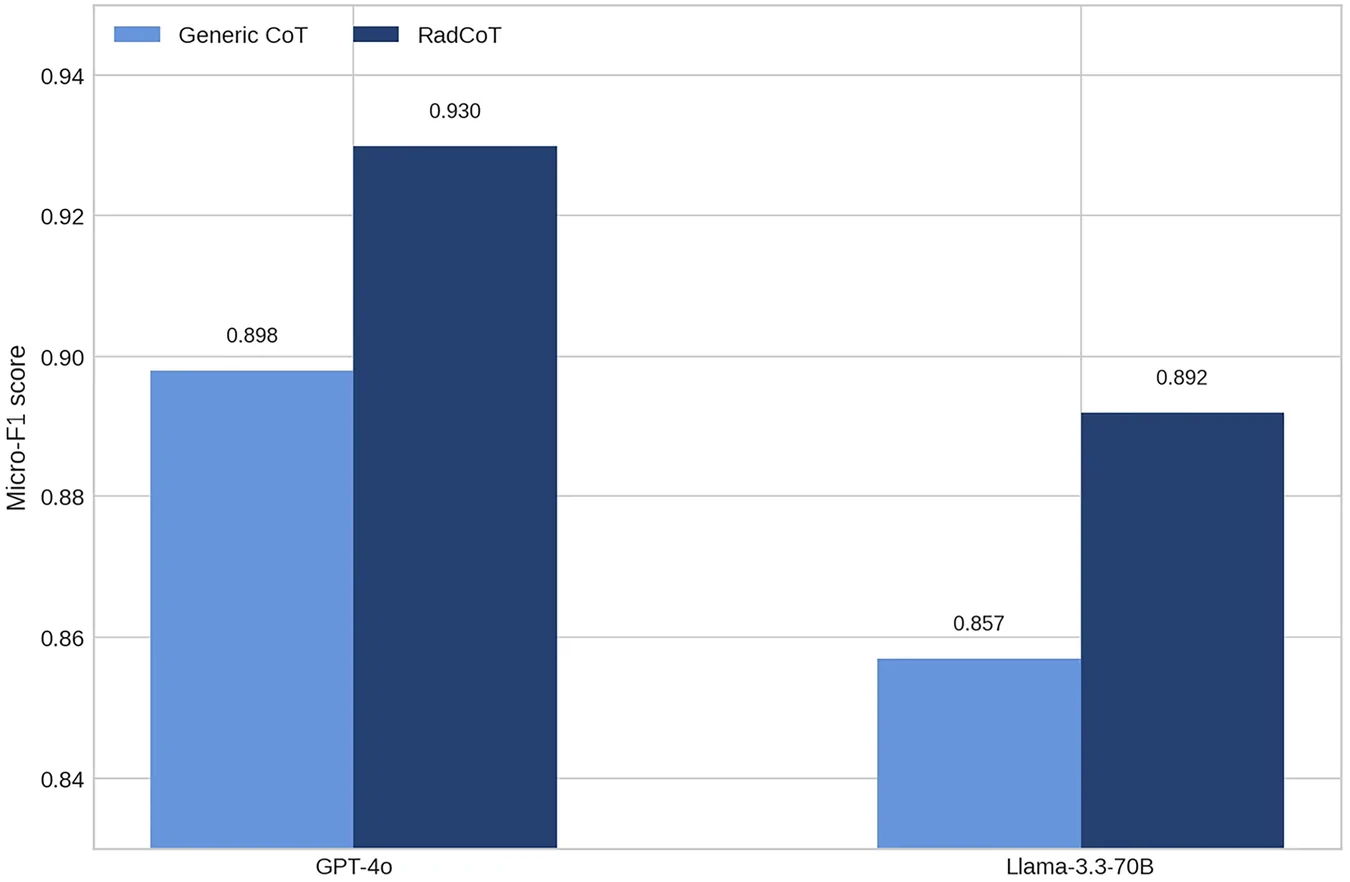
Sensitivity analysis of generic CoT *versus* RadCoT

To more directly assess whether the observed gains reflected radiology-specific prompt structure rather than stepwise prompting alone, we added a focused sensitivity analysis in GPT-4o and Llama-3.3-70B comparing a simple domain-neutral generic CoT prompt with RadCoT (Table 3 and Fig. 4). In both models, RadCoT outperformed generic CoT, although the magnitude of the gain differed across models. This pattern is consistent with the interpretation that generic stepwise prompting may improve performance, but that a radiology-specific scaffold may provide additional incremental benefit.

**Table 3:** Analysis comparing generic CoT and RadCoT

Model	Generic CoT Micro-F1	RadCoT Micro-F1	Absolute F1 gain of RadCoT over generic CoT	Relative F1 gain *versus* generic CoT	Generic CoT precision/recall	RadCoT precision/recall
GPT-4o	0.898	0.930	+0.032	+3.6%	0.913/0.884	0.941/0.920
Llama-3.3-70B	0.857	0.892	+0.035	+4.1%	0.868/0.847	0.903/0.881

### Error-type-specific performance

Error-type results are summarized in Table 4 and visualized in Fig. 3C. RadCoT yielded the largest absolute improvements for omission/insertion and interpretation errors, increasing mean F1 from 0.64 ± 0.04 to 0.82 ± 0.03 (Δ = 0.18, *p* = 0.006) and from 0.57 ± 0.04 to 0.75 ± 0.03 (Δ = 0.18, *p* = 0.008), respectively. Findings–impression discrepancies also improved substantially (0.69 ± 0.05 to 0.81 ± 0.04, Δ = 0.12, *p* = 0.012). By contrast, numerical and typographical errors had higher baseline performance under standard prompting (0.84 ± 0.03 and 0.74 ± 0.05), but still showed smaller yet significant gains with RadCoT (numerical to 0.89 ± 0.02, Δ = 0.05, *p* = 0.042; typographical to 0.83 ± 0.04, Δ = 0.09, *p* = 0.024).

**Table 4:** Summary performance of standard prompting *versus* RadCoT

Panel (A) Overall (aggregated across models)							
Metric	Standard	RadCoT		Δ	95% CI		*p*-value
Micro-averaged F1 (mean ± SD)	0.77 ± 0.06	0.85 ± 0.06		0.08	0.06–0.10		0.003
Precision (mean)	0.80	0.88		0.08	—		—
Recall (mean)	0.75	0.83		0.08	—		—
Inference time (relative)	1.00×	1.68×		68.20%	—		—
**Panel (B) Error-type-specific F1 (macro-average over models)**							
**Error type**	**Standard F1 (mean ± SD)**		**RadCoT F1 (mean ± SD)**		**ΔF1**	***p*-value**^**a**^	
Typographical	0.74 ± 0.05		0.83 ± 0.04		0.09	0.024	
Numerical	0.84 ± 0.03		0.89 ± 0.02		0.05	0.042	
Omission/insertion	0.64 ± 0.04		0.82 ± 0.03		0.18	0.006	
Interpretation	0.57 ± 0.04		0.75 ± 0.03		0.18	0.008	
Findings–impression discrepancy	0.69 ± 0.05		0.81 ± 0.04		0.12	0.012	
**Panel (C) Modality-specific F1 (aggregated across models)**							
**Modality**	**Standard F1**		**RadCoT F1**		**ΔF1**	***p*-value**	
Radiography	0.82		0.88		0.06	0.038	
Ultrasound	0.77		0.83		0.06	0.021	
CT	0.68		0.80		0.12	0.003	
MRI	0.66		0.78		0.12	0.004	

^a^ *p*-values are calculated using paired *t*-tests comparing Standard and RadCoT F1 scores

To assess likely deployment implications under more realistic clinical conditions, we performed a prevalence simulation using assumed report-error prevalences of 2%, 5%, 10%, and 20% (Table 5). Under these scenarios, the estimated positive predictive value increased from 0.145 at 2% prevalence to 0.676 at 20% prevalence, whereas the expected false-positive burden remained between 72.0 and 88.2 per 1,000 reports. These estimates suggest that, although negative predictive value remained high across scenarios, positive predictive value would decline substantially under lower-prevalence deployment conditions.

**Table 5:** Simulated deployment implications

Assumed error prevalence	PPV	NPV	False positives per 1000 reports	Missed error-containing reports per 1000 reports	Flagged reports per 1000 reports
2%	0.145	0.994	88.2	5.0	103.2
5%	0.305	0.986	85.5	12.5	123.0
10%	0.481	0.970	81.0	25.0	156.0
20%	0.676	0.936	72.0	50.0	222.0

## Discussion

### Principal findings

In this study, we developed RadCoT, a radiology-specific chain-of-thought prompting framework that aligns LLM reasoning with expert radiological review patterns. Across seven state-of-the-art LLMs and 1,170 real-world report errors, RadCoT consistently improved per-error F1 scores compared with standard prompting. Gains were largest for complex interpretive and inter-section discrepancies, precisely the error types traditionally regarded in the radiology QA literature as more clinically consequential than purely formal reporting defects [4], although a direct outcome-linked effect cannot be inferred from the present text-level data.

RadCoT enabled open-source models such as Llama-3.3-70B and Qwen-2.5-72B to approach the performance of commercial GPT-4o under standard prompting on this dataset, narrowing the performance gap while preserving the option for on-premises deployment. Whether this finding generalizes to other error distributions and clinical settings requires multi-institutional evaluation. This finding aligns with broader evidence that structured prompting and reasoning-oriented elicitation strategies can substantially improve LLM performance, including in medical domains [27, 34], and suggests a practical path toward privacy-preserving QA systems.

### Relationship to prior work

LLMs have been actively explored for clinical and documentation support tasks, and their performance is known to vary strongly with prompting strategy, task framing, and model family-often requiring careful prompt engineering to obtain reliable behavior in safety-critical settings [35]. General advances in chain-of-thought prompting and related stepwise elicitation methods have shown that explicitly structured reasoning can improve accuracy on complex multi-step tasks [25, 36]. Our results extend these insights to radiology report QA by introducing a structured CoT framework grounded in radiology practice, by systematically contrasting prompting strategies across a broad set of models and error categories.

At a methodological level, RadCoT builds on general advances in CoT prompting and on the growing literature demonstrating that LLMs can encode clinically relevant knowledge and benefit from medical-domain adaptation [34]. Our work adapts these insights to radiology by encoding the anatomy-centric, section-based, and inter-section logic of imaging reports directly into the prompt.

### Clinical implications and deployment considerations

From a clinical perspective, RadCoT is best viewed as a first-pass QA assistant rather than a replacement for human review. Even with RadCoT, complex interpretive errors—especially in CT and MRI—were missed in roughly one quarter of cases. A systematic error analysis (Supplementary Tables S3–S5) indicated that residual false negatives were concentrated in interpretation errors that required knowledge of external imaging criteria.

From a clinical deployment perspective, the prevalence simulation suggests that performance observed in our error-enriched evaluation set may overestimate real-world operational utility. Consistent with prior reports that errors are relatively infrequent in routine practice [1, 37], the projected PPV declined under lower-prevalence assumptions, indicating that some flagged reports in deployment would be false positives. Nevertheless, the estimated review burden remained potentially manageable for retrospective auditing or targeted pre-sign-off review, supporting the framework's practical relevance while underscoring that retrospective performance should not be interpreted as direct evidence of real-world clinical utility.

The 68% relative increase in inference time (absolute: ~7 s/report for GPT-4o; < 10 s for efficient open-source configurations) is compatible with retrospective auditing and pre-sign-off review, but is less suitable for real-time dictation, where latency-reduction strategies such as reasoning distillation or selective invocation on high-risk reports would be required. Importantly, AI-assisted review carries a well-documented risk of automation bias, whereby radiologists may uncritically accept model outputs or become desensitized to repeated false alarms; RadCoT should therefore be deployed with mandatory radiologist adjudication on every flagged item rather than as an autonomous filter.

Privacy, safety, and governance are central concerns for clinical adoption of LLMs [38, 39]. Our finding that open-source Llama 3.3 70B with RadCoT approximated the GPT-4o baseline performance suggests that institutions can implement strong QA systems while keeping both data and models on-premises, consistent with emerging guidance that emphasizes risk management, accountability, and context-specific controls for AI in healthcare. As a controlled methodological study, our corpus was intentionally error-enriched to enable stable performance estimation across error categories. Real-world radiology reporting involves substantially lower and more heterogeneous error prevalence, and performance in such settings remains to be validated. Nevertheless, the present study establishes a reliable methodological foundation—including the RadCoT prompting strategy, the five-category error taxonomy, and the evaluation protocol—upon which future prospective real-world studies can build.

Integration into existing workflows will require thoughtful user-interface design. Future prospective studies should evaluate how such tools affect reporting time, error rates, override patterns, and clinician satisfaction, building on established efforts in structured reporting and template-driven communication in radiology [17].

It is important to note that text-level QA has fundamental boundaries: RadCoT assesses internal consistency and adherence to reporting standards, but cannot detect perceptual misses at the image level, adjudicate diagnostic correctness against the underlying study, or assess clinical severity independently of outcome data. RadCoT is accordingly best positioned as a complementary first-pass layer within existing peer-review workflows, not as a replacement for image-level review. A definitive severity assessment requires integration with the clinical context, imaging findings, and downstream decision-making. Future prospective studies should incorporate outcome-linked severity grading (*e.g.*, by leveraging established peer-review frameworks such as the ACR RADPEER® scoring system [40] or institutional discrepancy classification schemes) to quantify the real-world clinical impact of detected *versus* missed errors.

### Limitations and future directions

This study has several limitations. First, the dataset was derived from a single-institution QA repository and may not fully capture the diversity of reporting styles, QA practices, and error spectra at other centers. Multi-institutional collaborations and public benchmark datasets will be important for assessing generalizability. Second, although our corpus of 1,170 errors is larger than many proof-of-concept LLM studies in clinical text settings, it remains modest relative to the scale of modern foundation models. Rare or highly nuanced error types may therefore be under-represented. In addition, the larger performance gain observed in CT/MRI than in radiography/ultrasound should be attributed unambiguously to report complexity alone, because an indirect advantage of RadCoT’s intersection cross-referencing and cross-sectional verification steps for multi-planar examinations cannot be excluded; disentangling these factors will require modality-stratified prompt-ablation studies in future work.

Third, errors were evaluated at the text level without direct integration of imaging data. While this mirrors current QA workflows that focus on the finalized report, the ultimate goal is multimodal systems that jointly reason over images and text. Recent progress in general-purpose biomedical multimodal modeling suggests that such joint reasoning is increasingly feasible, and combining RadCoT-like structured verification with multimodal models may further improve detection of subtle perceptual and interpretive errors.

Fourth, RadCoT relies on explicit reasoning traces, and chain-of-thought text is not necessarily a faithful representation of a language model’s internal computation [41, 42]. RadCoT is therefore best understood as a structured prompting scaffold whose utility is demonstrated through downstream error-detection performance, rather than as a mechanistic account of model decision-making. In clinical use, the reasoning trace serves as a human-verifiable rationale—each flagged error is displayed alongside the specific report span it refers to, allowing the reviewing radiologist to audit the flag against the source text regardless of trace faithfulness. Future work incorporating faithfulness probes (*e.g.*, counterfactual perturbation, truncation-based consistency checks) could further characterize this alignment.

Meanwhile, the analysis compares a simple domain-neutral generic CoT prompt with RadCoT in GPT-4o and Llama-3.3-70B. RadCoT, although shown to have an advantage over generic CoT in both models, suggests that the radiology-specific scaffold provides incremental value beyond generic stepwise reasoning alone. However, it should be noted that, to date, no universally accepted generic CoT benchmark has been developed for radiology report QA; this comparison should be interpreted as a focused analysis rather than a definitive mechanistic attribution.

## Conclusion

RadCoT, a radiology-specific chain-of-thought prompting framework, substantially improves LLM-based error detection in structured radiology reports, particularly for complex interpretive and inter-section discrepancies. The framework allows high-quality error detection with open-source models deployed on-premises, offering a practical and privacy-preserving path toward large-scale, AI-enabled QA in radiology.

## Supplementary information

Supplementary Material

Supplementary Table SP2

## Data Availability

The detailed prompt is accessible via the GitHub project provided at https://github.com/currylee92/Radcot-project.

## Abbreviation

*κ*: Cohen’s kappa
ACR: American College of Radiology
AI: Artificial intelligence
API: Application programming interface
AUROC: Area under the receiver operating characteristic curve
BERT: Bidirectional encoder representations from a transformer
CI: Confidence interval
CoT: Chain-of-thought
CT: Computed tomography
ESR: European Society of Radiology
F1: F1 score (harmonic mean of precision and recall)
FN: False negative
FP: False positive
GPT: Generative pre-trained transformer
GPU: Graphics processing unit
HTTPS: Hypertext Transfer Protocol Secure
IDPT: In-domain pre-training
IQR: Interquartile range
IRB: Institutional review board
LLM(s): Large language model(s)
MRI: Magnetic resonance imaging
NIST: National Institute of Standards and Technology
NLP: Natural language processing
P: Precision
QA: Quality assurance
R: Recall
RadCoT: Radiological chain-of-thought
RADPEER: Radiology peer review
TP: True positive
WHO: World Health Organization
